# Separating stroke and acute unilateral vestibulopathy using history, examination and vestibular tests: a machine learning approach

**DOI:** 10.1007/s00415-026-13779-0

**Published:** 2026-09-08

**Authors:** Chao Wang, Kunal Chaturvedi, Benjamin Nham, Nicole Reid, Andrew P. Bradshaw, Sally M. Rosengren, Deborah A. Black, Kendall J. Bein, G. Michael Halmagyi, Ali Braytee, Gnana K. Bharathy, Mukesh Prasad, Miriam S. Welgampola

**Affiliations:** 1 Central Clinical School, University of Sydney, Sydney, Australia; 2 Institute of Clinical Neurosciences, Royal Prince Alfred Hospital, Sydney, Australia; 3 School of Computer Science, Faculty of Engineering and Information Technology, University of Technology Sydney, Sydney, Australia; 4 St George and Sutherland Clinical School, University of New South Wales, Sydney, Australia; 5 Faculty of Medicine and Health, University of Sydney, Sydney, Australia; 6 Department of Emergency Medicine, Royal Prince Alfred Hospital, Sydney, Australia; 7 Royal Prince Alfred Green Light Institute for Emergency Care, Sydney, Australia

**Keywords:** Acute unilateral vestibulopathy, Vestibular neuritis, Stroke, Artificial intelligence, Machine learning

## Abstract

**Background:**

Acute unilateral vestibulopathy (AUVP) and posterior circulation stroke (PCS) are the two common causes of the acute vestibular syndrome. We developed and evaluated machine learning models to differentiate AUVP and PCS using combinations of patient history, examination, and vestibular tests.

**Methods:**

We recruited 294 patients presenting to the Emergency Room (ER) with acute vestibular syndrome (AUVP: *n* = 163, PCS: *n* = 131). Data from history, bedside examination and vestibular tests (video-nystagmography (VNG), video head-impulse test (VHIT), vestibular-evoked myogenic potentials (VEMP) and subjective visual horizontal) were used for machine learning model development. Different subsets of data simulated three scenarios hierarchically reflecting different levels of clinical expertise and resources: Tier 1 represented an ER with neuro-otology support (history, neuro-otological examination, VNG, VHIT, ocular VEMP), Tier 2 an ER with VHIT (history, basic examination, VHIT) and Tier 3 an ER reliant on history and basic examination only. Model performance was also compared against the HINTS test (head impulse, nystagmus, test-of-skew).

**Results:**

Our best-performing models used the CatBoost or XGBoost algorithms and identified PCS with accuracies of 96.6% (95% CI: 93.3–99.9%), 94.6% (95% CI: 90.5–98.6%) and 88.8% (95% CI: 86.0–91.6%) for Tiers 1, 2 and 3. HINTS by experts achieved 94.6% accuracy. The most important variables were the bedside head-impulse test, presence of focal neurological symptoms and spontaneous nystagmus slow phase velocity in Tier 1; focal neurological symptoms in Tier 2; and age in Tier 3.

**Conclusion:**

Machine learning models can accurately separate PCS and AUVP and hold promise as diagnostic aids for frontline clinicians.

**Supplementary Information:**

The online version contains supplementary material available at https://doi.org/10.1007/s00415-026-13779-0.

## Introduction

The acute vestibular syndrome (AVS) describes new-onset, severe and persistent vertigo and/or imbalance [1]. AVS constitutes 10–20% of all acute dizziness presentations to the Emergency Room (ER) and is usually due to either posterior circulation stroke (PCS) or acute unilateral vestibulopathy (AUVP), also known as vestibular neuritis [2]. Since stroke is life-threatening and necessitates urgent intervention, whilst AUVP is self-limiting and not life-threatening, it is important for clinicians to make a correct and timely diagnosis. However, AUVP and PCS are amongst the most misdiagnosed causes of dizziness in the ER, even by specialists [3].

To make their diagnostic decision, clinicians rely on the patient history, bedside examination and investigations including neuroimaging and audio-vestibular tests. The history is helpful if there are central neurological symptoms or new hearing loss/tinnitus, which are red flags for PCS [4]. Similarly, focal neurological abnormalities on examination suggest stroke. However, clinicians cannot rely on their presence as focal deficits may not be evident in two-thirds of PCS [2]. In the absence of obvious signs of PCS, the recommended approach is to then perform the 3-step HINTS examination (head impulse, nystagmus, test-of-skew) [5], which can exclude stroke with high accuracy if done by an expert. However, frontline clinicians who assess AVS are usually not experts at HINTS [6] and thus frequently avoid, incorrectly perform or misinterpret this valuable diagnostic algorithm [7, 8].

If the diagnosis remains uncertain after the examination, then investigations can help by providing objective evidence of pathology. If available, an MRI can confirm the diagnosis if it shows an acute stroke, but MRI is falsely negative in 15% of PCS when performed in the first 48 h [9]. Other useful investigations are the modern vestibular function tests. These include video-nystagmography (VNG), which can be used at the bedside to optimise nystagmus assessment [10], as well as tests that evaluate all 5 vestibular end-organs on both sides: the video head impulse test (VHIT) assesses the 3 semi-circular canals [11, 12], vestibular-evoked myogenic potentials (VEMPs) test the saccule (cervical VEMP) and utricle (ocular VEMP) [13] otolith organs, and the subjective visual horizontal (SVH) offers another way to evaluate utricular function [14].

**Fig. 1:**
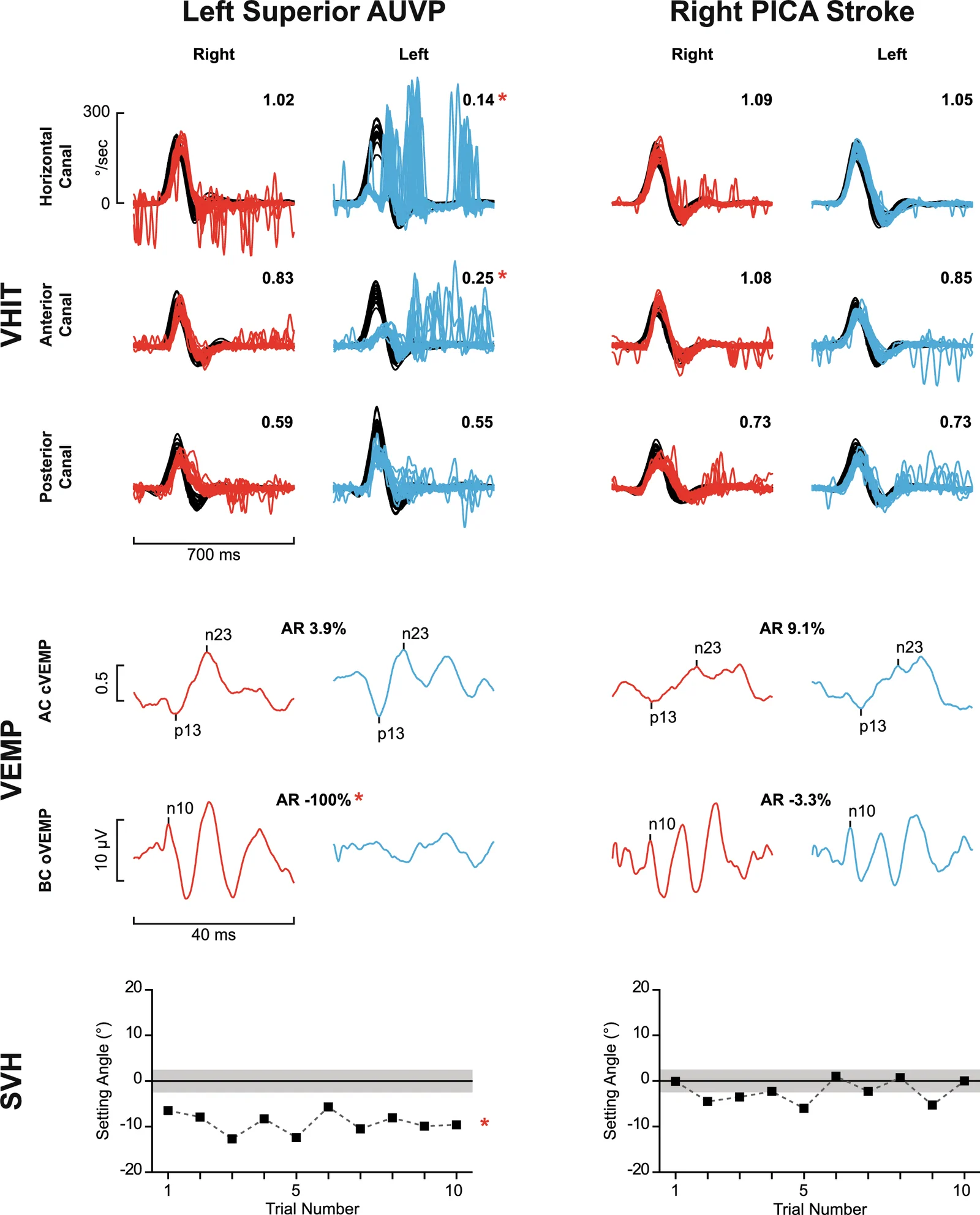
Vestibular function test results in acute unilateral vestibulopathy and posterior circulation stroke. Representative examples from our study cohort showing findings on VHIT, VEMPs and SVH in a patient with left superior vestibular AUVP and another with right PICA stroke. The AUVP patient has impairment (asterisks) on the left involving the horizontal and anterior semi-circular canals on VHIT and utricle on oVEMP and SVH. Conversely, the stroke patient’s results are all within normal limits. Red lines indicate right-sided results, blue lines indicate left-sided results. AC = Air-conducted, AR = Asymmetry ratio, AUVP = Acute unilateral vestibulopathy, BC = Bone-conducted, PICA = Posterior inferior cerebellar artery, SVH = Subjective visual horizontal, (c/o)VEMP = (cervical/ocular) vestibular-evoked myogenic potentials, VHIT = Video head impulse test

AUVP demonstrates characteristic profiles of deficits on these tests depending on which division(s) of the vestibular nerve are affected. In most cases, either the superior division (42–64%) or both divisions (33–56%) are affected, whilst the rare inferior AUVP only occurs in 2–5% [15, 16]. Superior AUVP may show deficits in the horizontal and anterior semi-circular canals on VHIT and the utricle on ocular VEMP and SVH (Fig. 1), whilst inferior AUVP only impairs the posterior canal on VHIT and saccule on cervical VEMP; all 5 end-organs may be impacted when both divisions are affected [16, 17]. Vestibular function testing that shows a typical pattern for AUVP helps confirm its diagnosis; conversely, as stroke can have diverse results on vestibular tests [18], there is no distinctive abnormality implying stroke, excepting that the results are incompatible with the alternate diagnosis of AUVP (Fig. 1).

**Fig. 2:**
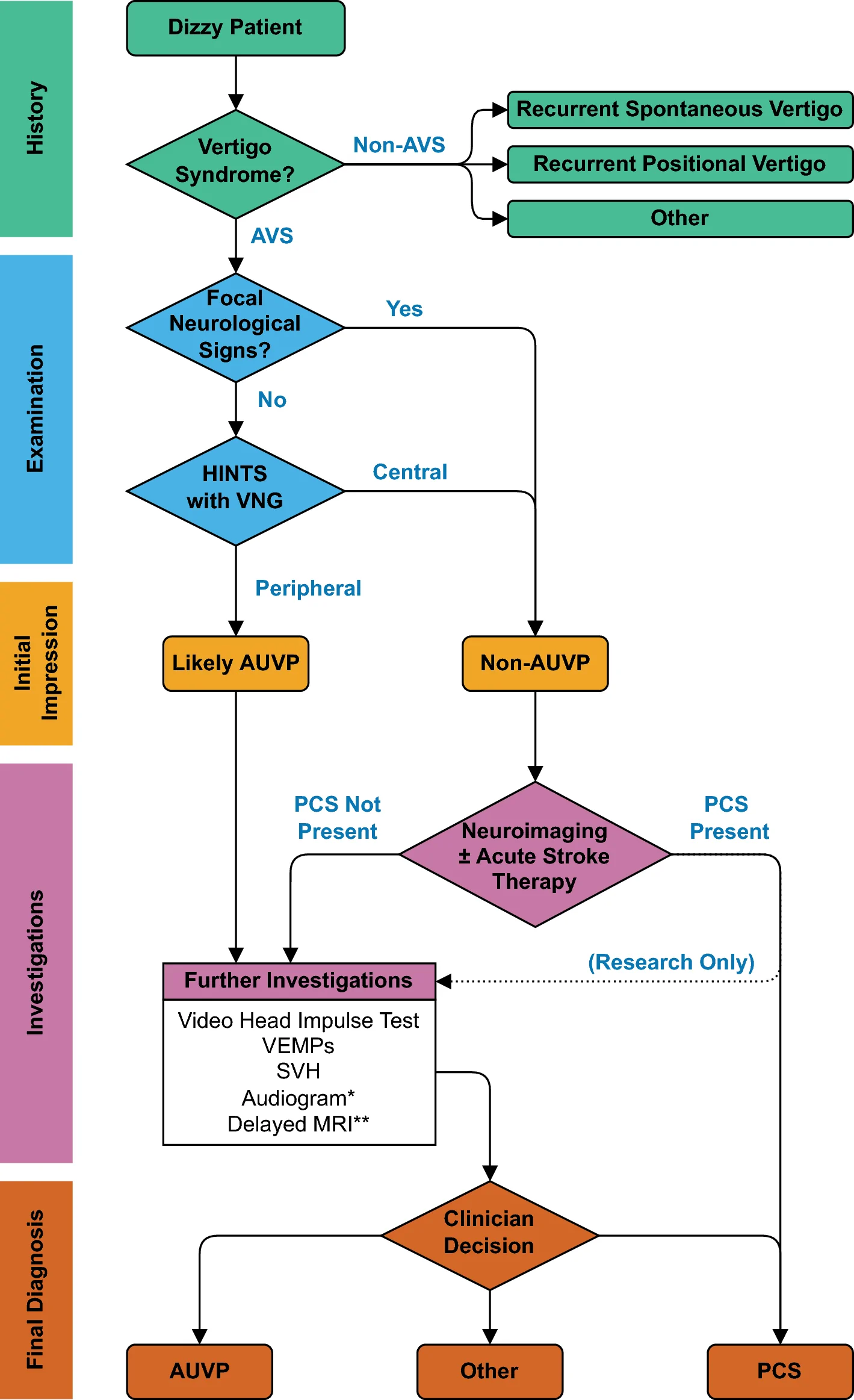
Diagnostic workflow for acute vestibular syndrome used by expert clinicians at our centre. HINTS was always performed with VNG at our centre. Additionally, patients with demonstrated PCS on neuroimaging also had vestibular function tests performed for research purposes (dotted arrow). *Audiometry was performed if AUVP was suspected. **Delayed MRI only performed for suspected AUVP if atypical findings present (see text). AUVP = Acute unilateral vestibulopathy, AVS = Acute vestibular syndrome, HINTS = Head Impulse, Nystagmus, Test-of-Skew, PCS = Posterior circulation stroke, SVH = Subjective visual horizontal, VEMPs = Vestibular-evoked myogenic potentials, VNG = Video-nystagmography

Not all Emergency Rooms have access to vestibular testing or experts trained in interpreting the results. Detailed audio-vestibular testing as illustrated in Fig. 1 may also be impractical due to the time-critical nature of acute stroke therapy. As shown in Fig. 2, tertiary referral centres may opt to use a combination of history, examination and high-yield investigations, in the interest of expediency.

Machine learning models have shown promise in differentiating vestibular disorders using clinical data [19–22]. Diagnostic models tailored to AVS may be able to enhance the diagnostic accuracy of specialists whilst also assisting clinicians without neuro-otology expertise. In this study, our objective was to develop machine learning models to classify AVS patients as PCS or AUVP using data from history, bedside examination and vestibular function tests. Additionally, our models could be adapted to diverse clinical scenarios by simulating different levels of clinician expertise and equipment availability using various subsets of training data.

## Methods

### Problem formulation

A collaboration of neuro-otologists, emergency doctors and data scientists addressed the problem of separating AUVP and PCS using machine learning models trained on data from history, examination and vestibular function tests. We used a standard data science lifecycle [23] consisting of problem formulation, data acquisition/preparation, model development and evaluation; the final steps of deployment and refinement were beyond the scope of this proof-of-concept study.

### Participants

Participants were consecutively recruited from adult patients who presented with sudden onset of vertigo or imbalance to the Emergency Room of Royal Prince Alfred Hospital, a major tertiary hospital with comprehensive stroke services in Sydney, Australia, between March 2018 and March 2023 and who were referred to the neurology service. These patients all underwent clinical assessment by a clinician with neuro-otology expertise (authors C.W., B.N., M.W., N.R. or G.M.H.) and were included if they met our diagnostic criteria for AUVP or PCS; this diagnosis served as the gold standard for machine learning purposes. The diagnosis of AUVP was made if they met all of the following criteria: (1) acute vertigo/imbalance present at rest for hours or more; (2) spontaneous unidirectional nystagmus on video-oculography; (3) either VHIT showing impaired gains (relative to our laboratory’s age-adjusted normative values [24]) in a pattern consistent with superior, inferior or pan-AUVP, or a positive bedside head impulse test by an experienced examiner with corresponding catchup saccades on VHIT (as AUVP can present as catchup saccades with normal gains on acute VHIT [25, 26]); (4) no diffusion restriction observed on MRI performed > 48 h postsymptom onset if skew was present, inferior AUVP was suspected or audiogram showed asymmetry with new hearing loss reported; and (5) not better accounted for by another diagnosis. PCS was diagnosed if CT and/or MRI imaging confirmed an acute ischaemic or haemorrhagic stroke in the posterior circulation. Patients were only included if reviewed within 7 days of symptom onset. Patients were excluded if they had a history of vestibulopathy that may confound assessment results, or if they were too unwell to participate in clinical assessment (e.g. reduced level of consciousness, delirium, or requiring intensive care).

### Data collection

Data from 203 variables collected from clinical assessment across the categories of history, bedside examination and 4 vestibular function tests (VNG, VHIT, VEMPs, SVH) were used to develop the machine learning models. The metadata for all variables is provided in Online Resource 1. These variables were chosen based on the expertise of 5 authors who frequently assess acute vestibular syndrome in the ER.

### History

We used 17 variables from demographics (age, sex) and a standardised history, consisting of categorical options for vertigo duration and the presence of spontaneous vertigo, positional vertigo, aural symptoms (namely hearing loss, tinnitus, aural fullness), headache, new focal neurological symptoms (which included any of diplopia, visual field deficit, dysphasia, dysarthria, ptosis, focal weakness, focal sensory deficits, and limb incoordination as reported by patients) and cardiovascular risk factors (hypertension, diabetes mellitus, hyperlipidaemia, atrial fibrillation, smoking, previous history of stroke or transient ischaemic attack, and family history of stroke).

### Bedside examination

The bedside examination was performed by an expert examiner and generated 9 variables based on whether any of the following were present: bi-directional gaze-evoked nystagmus, abnormal bedside head impulse test (including which side(s)), skew, abnormal saccades, abnormal pursuit, focal weakness, limb ataxia, truncal ataxia and gait ataxia.

### Vestibular function tests

As part of the clinical assessment, all patients were offered all 4 tests used by neuro-otologists to assess the peripheral vestibular organs: VNG, VHIT, VEMPs and SVH.

#### VNG

Video-nystagmography was used to examine with visual fixation removed for the dominant fast phase direction of any spontaneous, post head-shaking and positional nystagmus (on Dix-Hallpike and supine turn tests) present; additionally, the slow phase velocity (SPV) was calculated for spontaneous nystagmus using custom software [27]. Examination with visual fixation removed is best practice as the spontaneous nystagmus of AUVP can be partially or fully suppressed if visual fixation is present (nystagmus in PCS will also often suppress with fixation on VNG, but to a much lower degree) [10, 28]. Note that oculomotor assessment for gaze-evoked nystagmus, smooth pursuit and saccades was performed at the bedside without VNG.

#### VHIT

The VHIT was performed using the ICS Impulse system (Natus, CA, USA) for all 6 semi-circular canals. Custom software was chosen for VHIT analysis [29] to enable extraction of more detailed saccade metrics than the bundled software. This yielded 150 variables from VHIT. Each canal had 25 variables: the gain and the saccade characteristics, comprising the average saccade prevalence, amplitude, peak velocity, and duration for the first to fifth saccades (if present), as well as the average latency for the first saccade, total saccade prevalence, cumulative saccade amplitude and covert:all saccade ratio. VHIT was not performed in patients with demonstrated arterial dissection.

#### VEMPs

Cervical and ocular VEMPs were recorded in response to both air-conducted click and bone-conducted forehead tap stimuli using a Medelec Synergy device (version 20.0; Natus, CA, USA) as per previously published methods [30]. For each combination of VEMP type and stimulus type, there were 5 variables (amplitude and latency for right and left sides, as well as the asymmetry ratio between the sides), resulting in a total of 20 VEMP variables.

#### SVH

The SVH was performed by asking patients to use a handheld controller to align an illuminated bar to their perceived gravitational horizon, whilst seated upright in a dark room 1.5 m away with their head immobilised [31]. Each test consisted of 10 trials with random starting angles, and the mean offset of the last 5 trials was taken as the single variable from this test.

### Classification by machine learning models

**Fig. 3:**
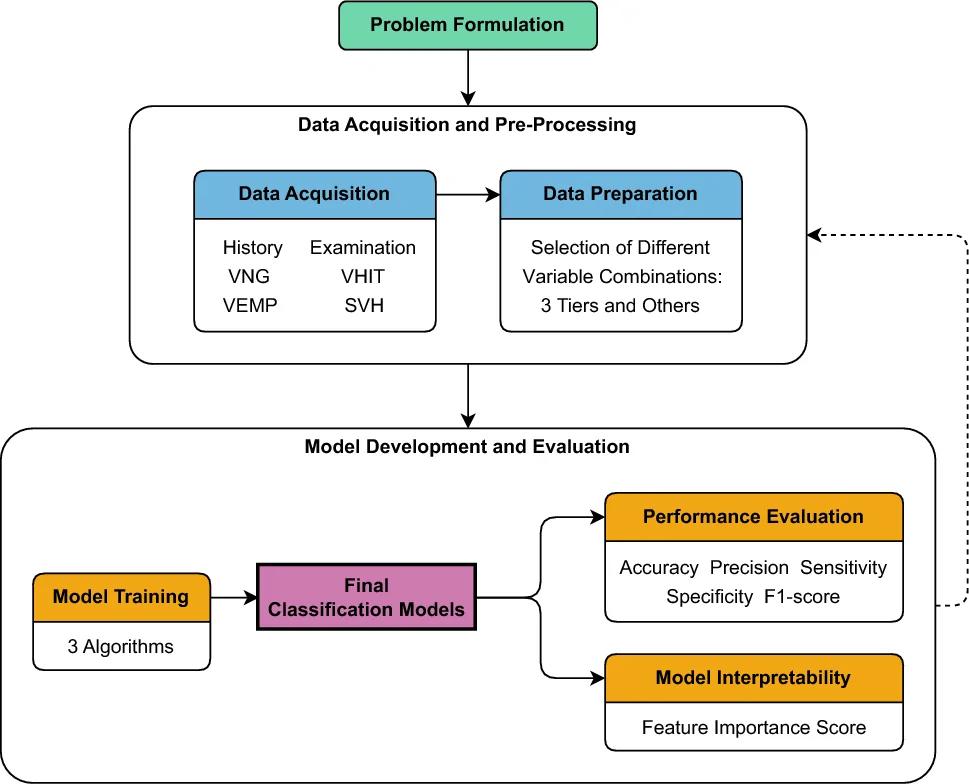
Workflow for machine learning model development and evaluation. The dotted arrow indicates that this was an iterative process. SVH = Subjective visual horizontal, VEMP = Vestibular-evoked myogenic potentials, VHIT = Video head impulse test, VNG = Video-nystagmography

We used the scikit-learn Python machine learning library version 1.5.2 [32] to develop and evaluate classification models that labelled patients as AUVP or PCS; our workflow is shown in Fig. 3. Although we did consider models that used all 203 available variables, we recognised that requiring every variable would be impractical in the clinical setting, particularly as clinician expertise and vestibular testing may be unavailable. Accordingly, we focussed on developing separate machine learning models that were tailored for clinicians in 3 different scenarios reflecting various levels of expertise and resources. This was done by restricting the data available for model development to different “tiers” that included only what was readily available and likely to be helpful in each scenario.

The first combination of data (“Tier 1”) simulated an Emergency Room supported by a comprehensive neuro-otology service, and included the history, full neuro-otological examination, VNG, VHIT from the two horizontal canals only and bone-conducted ocular VEMPs (our earlier work indicated that these tests were most effective for separating AUVP and PCS [18]). A second combination (“Tier 2”) represented a non-expert ER clinician who is unfamiliar with the more advanced components of the bedside examination, but is proficient in a basic neurological examination and has access to VHIT; this tier’s data were restricted to the history, a limited basic examination (focal weakness and limb/truncal/gait ataxia only; notably, the bedside head impulse test and skew were excluded) and horizontal canal VHIT. A third combination (“Tier 3”) was for the same clinician as in Tier 2 but without access to VHIT, and thus only used the history and same limited basic examination data. Online Resource 2 provides the full list of variables used by each tier. Finally, we also explored the utility of each data category individually for separating AUVP and PCS using machine learning, by training models on datasets consisting solely of history, examination (both neuro-otological and basic), VNG, VHIT, VEMP or SVH variables.

For each tier of available data, we trialled 3 boosted tree-based machine learning algorithms—XGBoost [33], LightGBM [34] and CatBoost [35]—as the optimal algorithm for a given dataset is unknown prior to modelling. Boosted tree algorithms were chosen due to their demonstrated effectiveness in classifying vestibular disorders using similar clinical data [19, 20, 36] as well as their efficiency [37] and interpretability [38]. For the Tier 3 dataset, we also created a machine learning model that used a logistic regression algorithm [39] as a point of comparison against a simpler algorithmic approach.

For data pre-processing, the variables were prepared appropriately prior to being used as input to the machine learning models to ensure compatibility and optimal performance. Categorical responses from history and examination were converted to the categorical data type (or to numerical using one-hot encoding for the logistic regression model), and ordinal variables, such as the duration of vertigo, were encoded according to their inherent ranking. A total of 7.8% of the dataset values were missing. For all 3 boosted tree-based algorithms, missing values were retained as is, given the inherent capability of the algorithms to handle missing data. For the logistic regression model, missing numerical values were imputed with the median and missing categorical values imputed with the most frequent category; numerical features were also standardised with StandardScaler.

Default algorithm parameters were used. To avoid data inefficiency, bias and variability, we used 5-fold stratified cross-validation to evaluate the models developed with each data combination on the metrics detailed in Statistical Analysis below; this method maximises data usage and produces more reliable results [40]. The same seed value was used during model development to allow comparison between algorithms and tiers.

To gain insights into which variables had the greatest impact on model predictions, we extracted feature importance scores from the algorithms (“feature” is another term for variable in machine learning) for the best-performing model within each tier, as well as for selected datasets (namely the all data, history variables only and VHIT variables only datasets). Feature importance scores quantify how influential each variable is in contributing to the model’s predictive decisions. Specifically, these scores were extracted based on split frequency for XGBoost and LightGBM [33, 34] and prediction value changes for CatBoost [35], with higher scores indicating variables that significantly enhance model accuracy and predictive performance [33, 38].

### Classification by HINTS

As a point of comparison, we evaluated how HINTS performed on the same metrics when applied to the same evaluation sets that the models were assessed on during 5-fold cross-validation. The prediction from HINTS was determined for each patient by applying the following rules to their data: the diagnosis was AUVP if there was no vertical component of spontaneous nystagmus, no bi-directional gaze-evoked nystagmus, no skew and either a) left-beating spontaneous nystagmus with a positive rightward bedside head impulse test or b) right-beating spontaneous nystagmus with a positive leftward bedside head impulse test; if this was not met, then the patient was labelled PCS. Finally, we compared HINTS against a version without the test-of-skew, that only used the head impulse and nystagmus results.

### Evaluation metrics and statistical analysis

The machine learning models and HINTS were both evaluated using the following metrics (“performance metrics”): accuracy, precision, sensitivity, specificity and F1-score (which is the harmonic mean of precision and recall (sensitivity)). PCS was defined as the positive diagnosis. We used the default probability cut-off of ≥ 0.50 to identify PCS. For the models, we calculated 95% confidence intervals using the 5 iterations generated by cross-validation. Due to relatively balanced representation of both classes, accuracy was chosen as the primary metric.

Additionally, we compared the performance of the best machine learning model in each tier, the logistic regression model, and HINTS using McNemar’s test on the proportion of incorrect predictions [41], aggregated across the 5 cross-validation folds. Statistical analysis was performed using R (version 4.4.0), and statistical significance was defined as *p* ≤ 0.05.

### Sample size calculation

We used all available collected data for model development. However, we estimated the sample size required to separate AUVP and PCS using machine learning via a surrogate VHIT parameter (as VHIT features in most tiers of data availability). Based on earlier work [18], first saccade duration was conservatively chosen out of several VHIT parameters as it would require the largest sample size to separate AUVP and PCS. We calculated using its published value and a PCS:AUVP enrolment ratio of 0.60 [42] that 80% power would be achieved by a sample size of 26 AUVP and 16 PCS patients, and this was exceeded by our dataset.

## Results

Data were collected from 294 patients, 163 (55.4%) with AUVP and 131 (44.6%) with PCS. Patient characteristics are shown in Table 1.

**Table 1:** Patient characteristics

	Acute unilateral vestibulopathy (n = 163)	Posterior circulation stroke (*n* = 131)
History		
Age at clinical presentation, years, median (IQR)	59 (40–70)	66 (60–76)
Sex, female, n (%)	64 (39.3)	47 (35.9)
Spontaneous vertigo, n (%)	159 (97.5)	95 (72.5)
Duration of vertigo, n (%)		
No vertigo	0 (0)	26 (19.8)
Second(s)	0 (0)	4 (3.1)
Minute(s)	2 (1.2)	9 (6.9)
Hour(s)	24 (14.7)	27 (20.6)
Day(s)	133 (81.6)	62 (47.3)
One week	4 (2.5)	3 (2.3)
Subjective hearing loss, n (%)		
None	130 (79.8)	95 (72.5)
Pre-existing	28 (17.2)	24 (18.3)
New	5 (3.1)	12 (9.2)
Tinnitus, n (%)		
None	133 (81.6)	118 (90.1)
Pre-existing	14 (8.6)	9 (6.9)
New	16 (9.8)	4 (3.1)
Headache, n (%)	47 (28.8)	40 (30.5)
Focal neurological symptoms, n (%)	5 (3.1)	86 (65.6)
Number of cardiovascular risk factors, median (IQR) ^a^	1 (0–2)	2 (1–3)
Examination		
Spontaneous nystagmus dominant direction, n (%)		
No nystagmus	2 (1.2) ^b^	71 (54.2)
Left-beating	78 (47.9)	24 (18.3)
Right-beating	80 (49.1)	19 (14.5)
Up-beating	0 (0)	5 (3.8)
Down-beating	2 (1.2)	5 (3.8)
Mixed horizontal/vertical	1 (0.6)	6 (4.6)
Pure torsional	0 (0)	1 (0.8)
Spontaneous slow phase velocity, °/s, median (IQR)	11.1 (6.8–15.7)	0.0 (0.0–4.6)
Gaze-evoked nystagmus, n (%)	0 (0)	33 (25.2)
Bedside head impulse test, n (%)		
Normal	3 (1.8)	98 (74.8)
Abnormal to left	81 (49.7)	10 (7.6)
Abnormal to right	78 (47.9)	6 (4.6)
Abnormal bilaterally	1 (0.6)	9 (6.9)
Equivocal	0 (0)	3 (2.3)
Not performed ^c^	0 (0)	5 (3.8)
Skew, n (%)	2 (1.2)	24 (18.3)
Focal weakness, n (%)	1 (0.6)	24 (18.3)
Limb ataxia, n (%)	4 (2.5)	46 (35.1)
Truncal ataxia, n (%)	2 (1.2)	22 (16.8)
Gait ataxia, n (%)	47 (28.8)	80 (61.1)

^a^ Cardiovascular risk factors included hypertension, diabetes mellitus, hyperlipidaemia, atrial fibrillation, smoking, previous stroke/transient ischaemic attack and family history of stroke

^b^ Spontaneous nystagmus with visual fixation removed was not present in 2 acute unilateral vestibulopathy patients at initial review, but was present with visual fixation in one and present the following day in the other

^c^ Bedside head impulse test not performed in 5 stroke patients due to acute dissection (4) or severe horizontal ophthalmoplegia (1)

### Classification by machine learning models

#### Model performance across tiers

The best-performing Tier 1 model (using data from history, neuro-otological bedside examination, VNG, horizontal canal VHIT and bone-conducted ocular VEMPs) used the CatBoost algorithm and identified PCS with 96.6% accuracy (95% CI: 93.3–99.9%).

In Tier 2 (using history, basic bedside examination and horizontal canal VHIT metrics), CatBoost again yielded the best model which achieved 94.6% accuracy (95% CI: 90.5–98.6%).

For Tier 3 models (using only history and basic bedside examination), XGBoost had the highest accuracy of 88.8% (95% CI: 86.0–91.6%).

Table 2 shows the full performance metrics for models using all 3 algorithms in all 3 tiers of data availability. The models performed reasonably well across all algorithms and tiers: accuracies ranged from 86.7 to 96.6%, and there were similarly strong results in all the other metrics with all values 81.7% or more.

**Table 2:** Performance metrics of classification methods for separating posterior circulation stroke and acute unilateral vestibulopathy

	Accuracy	Precision	Sensitivity	Specificity	F1-score
Tier 1 Models using history, neuro-otological examination, VNG, horizontal canal VHIT and BC ocular VEMPs					
CatBoost	96.6 (93.3–99.9)	97.7 (95.0–100.0)	94.7 (88.3–100.0)	98.1 (96.0–100.0)	96.1 (92.2–100.0)
XGBoost	95.2 (91.5–99.0)	96.1 (91.3–100.0)	93.1 (86.1–100.0)	96.9 (93.1–100.0)	94.5 (90.0–99.0)
LightGBM	94.9 (90.0–99.8)	94.0 (88.7–99.3)	94.7 (87.0–100.0)	95.1 (90.7–99.4)	94.3 (88.7–99.9)
Tier 2 Models using history, basic bedside examination and horizontal canal VHIT					
CatBoost	94.6 (90.5–98.6)	92.8 (86.7–98.8)	95.4 (89.2–100.0)	93.9 (88.5–99.2)	94.0 (89.5–98.5)
LightGBM	93.2 (90.6–95.8)	91.5 (84.9–98.0)	93.9 (89.6–98.2)	92.6 (86.3–98.9)	92.5 (89.9–95.1)
XGBoost	90.8 (87.0–94.7)	88.8 (80.4–97.1)	91.6 (85.3–97.8)	90.2 (81.9–98.4)	89.9 (86.0–93.8)
Tier 3 Models using history and basic bedside examination					
XGBoost	88.8 (86.0–91.6)	88.3 (85.5–91.2)	86.3 (80.1–92.5)	90.8 (87.9–93.6)	87.2 (83.8–90.7)
CatBoost	88.1 (83.9–92.3)	89.8 (82.1–97.4)	83.2 (75.2–91.2)	92.0 (85.5–98.5)	86.1 (81.1–91.2)
LightGBM	86.7 (82.7–90.8)	88.0 (81.0–95.0)	81.7 (76.8–86.7)	90.7 (84.5–96.9)	84.7 (80.6–88.7)
Models using all data					
CatBoost	96.6 (92.7–100.0)	98.4 (94.0–100.0)	93.9 (88.5–99.3)	98.8 (95.4–100.0)	96.1 (91.5–100.0)
XGBoost	95.2 (92.1–98.4)	96.1 (92.5–99.6)	93.1 (87.9–98.4)	97.0 (94.3–99.6)	94.5 (90.9–98.2)
LightGBM	94.9 (91.0–98.8)	94.1 (89.1–99.1)	94.7 (87.8–100.0)	95.1 (90.6–99.5)	94.3 (89.8–98.8)
Models using history only					
XGBoost	86.1 (81.1–91.1)	85.4 (78.4–92.4)	83.2 (74.8–91.7)	88.3 (82.1–94.6)	84.1 (78.4–89.9)
CatBoost	84.7 (79.8–89.6)	86.7 (80.0–93.4)	77.8 (66.3–89.3)	90.2 (84.4–95.9)	81.7 (74.7–88.7)
LightGBM	84.3 (81.5–87.2)	84.2 (76.4–92.0)	80.9 (72.1–89.7)	87.0 (78.6–95.5)	82.2 (79.3–85.0)
Models using VHIT only					
CatBoost	89.5 (81.3–97.7)	87.9 (79.0–96.8)	88.5 (76.8–100.0)	90.2 (82.8–97.5)	88.1 (78.5–97.7)
XGBoost	89.1 (81.5–96.8)	89.9 (79.2–100.0)	85.5 (77.6–93.4)	92.0 (83.4–100.0)	87.6 (78.8–96.3)
LightGBM	86.4 (79.0–93.8)	85.5 (77.1–94.0)	83.9 (71.0–96.8)	88.3 (80.5–96.2)	84.4 (75.3–93.6)
HINTS	94.6 (90.8–98.3)	92.8 (86.6–99.0)	95.5 (90.5–100.0)	93.9 (88.5–99.2)	94.0 (89.9–98.2)
HIN	94.2 (91.8–96.6)	94.0 (88.8–99.2)	93.2 (89.4–97.0)	95.1 (90.8–99.4)	93.5 (90.9–96.1)

Machine learning models are labelled by the algorithm used for their development and sorted within each tier of data availability by descending accuracy. Values are % (95% confidence interval). F1-score is the harmonic mean of precision and recall (sensitivity). Neuro-otological examination includes all examination variables collected. Basic bedside examination only includes weakness and limb, truncal and gait ataxia. BC = Bone-conducted, HIN = Head impulse and nystagmus (only), HINTS = Head impulse, nystagmus, test-of-skew, VEMPs = Vestibular-evoked myogenic potentials, VHIT = Video head impulse test, VNG = Video-nystagmography

The performance of the best Tier 1 model was not significantly different from that of the best Tier 2 model (*p* = 0.13), and both outperformed the best Tier 3 model (*p* < 0.001 and *p* = 0.003 respectively). The best Tier 3 model was significantly superior to the logistic regression model that used the same Tier 3 dataset (*p* = 0.02), which reached an accuracy of 84.7% (95% CI: 79.8–89.6%) (see Online Resource 3 for full metrics).

The best model which used all collected data had the same accuracy as the best Tier 1 model (96.6%) and performed similarly across the other metrics. Interestingly, excluding ocular VEMP data from Tier 1 still resulted in the same accuracy as the best Tier 1 model. Another noteworthy result was that substituting the VHIT metrics in the Tier 2 model for VNG metrics (for spontaneous nystagmus only) yielded in similar performance, including 93.9% accuracy (95% CI: 90.3–97.4%) (Online Resource 3).

#### Models using individual data categories

When models using individual data categories were compared, the neuro-otological examination was most effective at separating PCS and AUVP (accuracy 94.6%). VHIT was next with 89.5% accuracy (dropping to 88.1% if only the gains were used), followed by VNG (87.4% accuracy). A model using only history reached 86.1% accuracy, whilst models using basic bedside examination (74.1% accuracy), SVH (70.4% accuracy) and VEMPs (69.4% accuracy) did not perform well. Table 2 shows the full performance metrics for the models developed using all data, history data only and VHIT data only; the performance metrics for all algorithms and data subsets trialled are available in Online Resource 3. CatBoost appeared to be the best algorithm overall, followed by XGBoost.

#### Variable importance

**Fig. 4:**
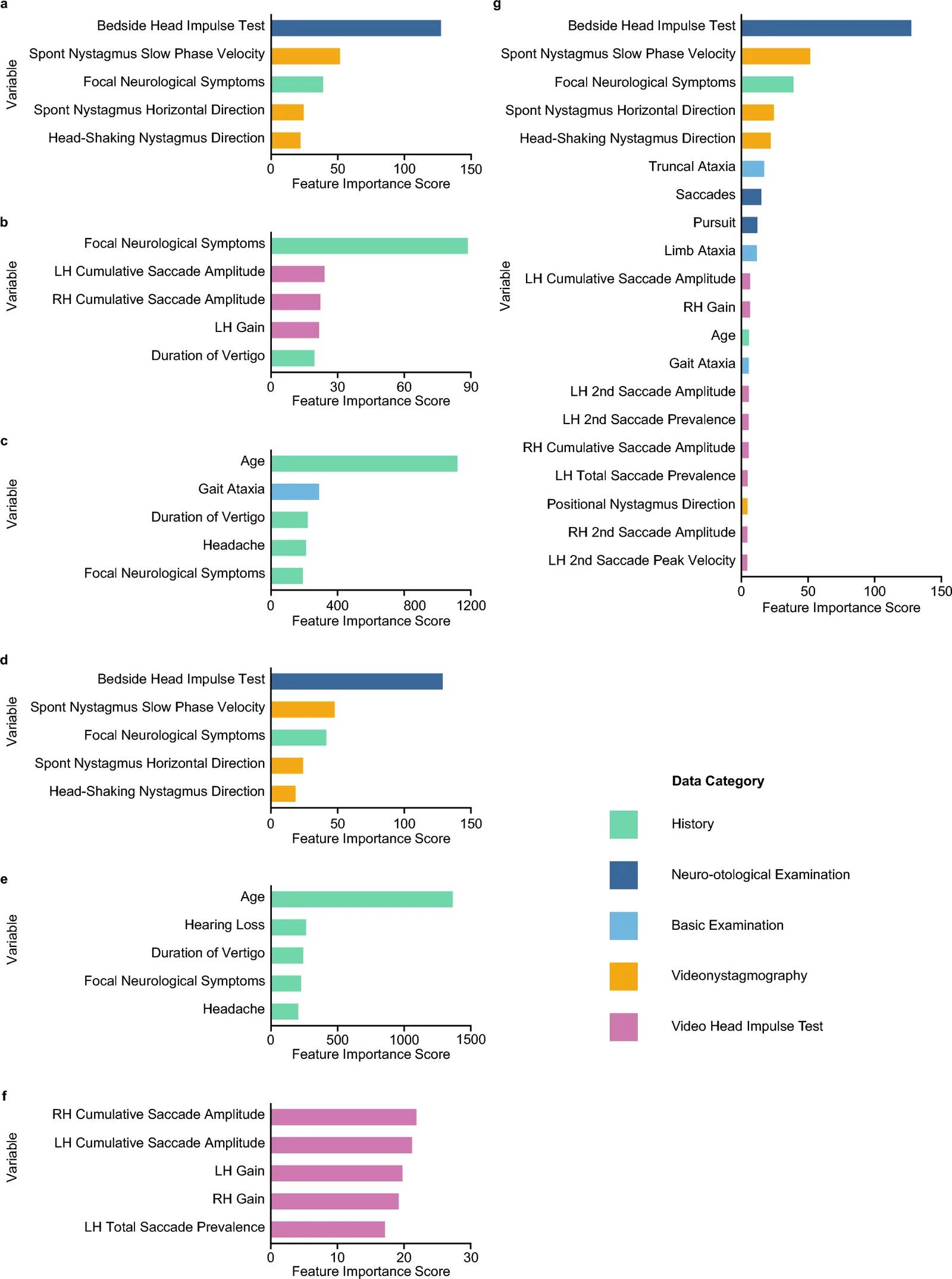
Variables with highest feature importance scores for the best models using different combinations of available data. Parts a-f show the top 5 variables for each combination: a Tier 1 (CatBoost): history, neuro-otological examination, video-nystagmography, horizontal canal VHIT, bone-conducted ocular VEMPs; **b** Tier 2 (CatBoost): history, basic examination, horizontal canal VHIT; **c** Tier 3 (XGBoost): history and basic examination. **d** All data (CatBoost); **e** history only (XGBoost); and **f** VHIT only (CatBoost). Part **g** shows the top 20 variables for Tier 1. A higher score indicates greater importance of the variable for model prediction. Colours indicate the data category which the variable is from. LH = Left horizontal canal, RH = Right horizontal canal, Spont = Spontaneous, VEMPs = Vestibular-evoked myogenic potentials, VHIT = Video head impulse test

For the best model in each tier as well as models using all data, history only and VHIT only, ranking the variables by feature importance scores identified which ones had the most influence on that model’s predicted diagnosis (Fig. 4 shows the top 5 for each model and the top 20 for Tier 1; the top 20 for Tiers 2 and 3 are shown in Online Resource 4). For the best Tier 1 and all data models, the bedside head impulse test was the variable with the highest importance, followed by the spontaneous nystagmus slow phase velocity and then the presence of focal neurological symptoms on history. For the best Tier 2 model, the presence of focal neurological symptoms was the most important variable, whilst age was most influential in the best Tier 3 and history only models. No variable stood out in the VHIT only model, but the gain and cumulative saccade amplitude for both the left and right horizontal canals were amongst the top 5.

#### Performance in a typical cases

We analysed the performance of our tiered models in atypical cases of AUVP and PCS, which present a greater diagnostic challenge for clinicians. There were 2 inferior AUVP patients, and 2 AUVP patients with skew (these all had a delayed MRI to exclude stroke). In both these groups, one case was correctly diagnosed and the other incorrectly diagnosed by the best model in all 3 tiers. HINTS made the same predictions for the inferior AUVP patients, but misdiagnosed both AUVP patients with skew. There were also 2 AUVP patients who did not have spontaneous nystagmus captured on VNG on presentation (see Table 1 for further details); both of these were correctly classified by the best model in all 3 tiers but misdiagnosed by HINTS. Finally, we considered the 18 PCS patients who had a unilaterally reduced horizontal canal gain on VHIT, as per our laboratory’s normal values. Sixteen were correctly categorised by models in all 3 tiers, whilst 2 were given the correct diagnosis by only one tier (Tier 2 for one and Tier 3 for the other).

#### Patients misclassified by models

Of the patients, 85% were corrected classified by the best model in all 3 tiers, 10% by models in 2 tiers, 3% by a model in one tier only, and 1% were incorrect classified in all 3 tiers. The 1% (3 patients) that all models misclassified consisted of the 2 atypical cases of AUVP discussed above (one inferior AUVP and one with skew) and a pontine stroke with no focal neurological signs and no nystagmus but with a unilaterally abnormal bedside head impulse test. Among the other patients misclassified by the best Tier 1 model, the majority were PCS patients, of whom all bar one had a purely horizontal spontaneous nystagmus and an abnormal bedside head impulse test. The other patients misdiagnosed by the best Tier 2 and 3 models included PCS patients with no focal neurological symptoms and/or (for Tier 2) abnormal VHIT gains, as well as AUVP patients with focal neurological symptoms and/or several cardiovascular risk factors and/or (for Tier 2) relatively preserved VHIT gains.

#### Effect of changing probability threshold

Finally, although for the purposes of this study we used a default probability cut-off of ≥ 0.50 to identify PCS, we acknowledge that in practice clinicians may prefer a lower cut-off given the higher cost of missing PCS. To characterise the effect of this, we repeated modelling for the best model in each tier to compare performance across 3 cut-offs of ≥ 0.10, ≥ 0.25 and ≥ 0.50. As expected, lower cut-offs improved sensitivity for PCS at the cost of specificity (e.g. the ≥ 0.10 cut-off yielded sensitivities of 99.3%, 100% and 95.4% for tiers 1, 2 and 3 with specificities of 88.9%, 80.3% and 77.3%); full tabulated results are available in Online Resource 5.

### Classification by HINTS

When evaluated in the same manner 5-fold manner as the machine learning models, HINTS identified PCS with 94.6% accuracy, 95.5% sensitivity and 93.9% specificity. Applying the principles of HINTS to just the nystagmus direction and bedside head impulse test (i.e. omitting the test-of-skew component) achieved 94.2% accuracy, 93.2% sensitivity and 95.1% specificity. Full performance metrics for both methods are shown in Table 2. On statistical analysis, the performance of HINTS was not significantly different from that of the best Tier 1 (*p* = 0.16) and Tier 2 models (*p* > 0.99), but was superior to that of the best Tier 3 model (*p* = 0.008).

## Discussion

In our study, we developed machine learning models for classifying AVS patients as posterior circulation stroke or acute unilateral vestibulopathy using data from history, examination and vestibular function tests. We tailored our models for clinicians in 3 different settings by using limited datasets compatible with each. Our models separated PCS and AUVP with high accuracy, precision, sensitivity, specificity and F1-score in each tier of data availability, including models designed for non-expert clinicians.

### Model performance and variable importance across tiers

Among the machine learning models, although the best Tier 1 model (simulating an ER supported by a specialist neuro-otology service) had a slightly higher accuracy than the best Tier 2 model (representing a clinician confident in basic bedside neurological examination for weakness and ataxia and supported by VHIT), their difference in performance was not statistically significant. This is a promising result, as the Tier 2 model is more accessible due to requiring only basic examination skills and less equipment. Our studies will need to be confirmed in future studies using larger datasets. Both the best Tier 1 and 2 models significantly outperformed the best Tier 3 model (designed for non-expert clinicians using history and basic examination only), which is unsurprising given the very limited data available to this model. Despite this, the best Tier 3 model still performed well with an accuracy of 88.8%; we are cautiously optimistic about this result given that history and examination skills are variable. Our Tier 3 model was superior to a simple logistic regression model using the same data, suggesting that more sophisticated machine learning algorithms offer a diagnostic advantage.

On variable importance analysis, the bedside head impulse test, spontaneous nystagmus SPV and presence of focal neurological symptoms were the 3 most influential variables in Tier 1. Their usefulness is expected, as the former is the most useful component of HINTS [2], SPV significantly differs between AUVP and PCS [28], and focal neurological symptoms are indicative of PCS. However, the bedside head impulse test is difficult for non-experts, whilst nystagmus SPV requires specialised software and equipment, so neither are accessible to non-expert clinicians. In Tier 2, which did not use the bedside head impulse test or SPV, focal neurological symptoms were by far the most influential, but several VHIT metrics were also important and may have functioned as a substitute for the bedside head impulse test. Focal neurological symptoms also feature in the top 5 variables for the best Tier 3, all data and history only models. The importance of focal neurological symptoms in our models likely reflects the fact that in our dataset 65.6% of PCS patients reported focal neurological symptoms compared to only 3.0% of AUVP. The most influential variable for the Tier 3 and history only models was age, a recognised risk factor for stroke [43]. The duration of vertigo also featured in the top 5 for several datasets, presumably as patients with “classic” stroke symptoms are likely to present sooner to ER and thus have an overall shorter duration of vertigo at assessment.

Given that the most influential variables were largely consistent with clinical expectations, it was unsurprisingly that patients with atypical clinical features accounted for a substantial proportion of the misclassified cases in each tier. Despite this, our analysis of the atypical subgroups of AUVP and PCS specified above indicates that our models may still offer diagnostic value for these cases, including AUVP patients who would otherwise fail HINTS; validation on a much larger dataset is essential given the very limited patient numbers in these subgroups.

### Performance of HINTS

HINTS achieved 94.6% accuracy. Notably, the best Tier 2 model, which did not use any HINTS components but did have access to horizontal canal VHIT, had the same accuracy as HINTS and there was no significant difference in the performance of these two methods. This indicates that VHIT may be an adequate substitute for clinicians who are not confident with HINTS. Omitting the test-of-skew from HINTS resulted in similar overall accuracy but higher specificity for PCS at a cost of lower sensitivity, an expected result given skew was included in the HINTS paradigm to improve sensitivity for brainstem strokes [5].

### Models using individual data categories and other data subsets

The accuracy of HINTS matched that of the model using the neuro-otological examination, affirming that HINTS captures the key diagnostic components of the full examination. However, an expert examination is not essential for effective separation of AUVP and PCS, as this was also achieved by models using only VHIT data (89.5% accuracy) or VNG data (87.4% accuracy) which may offer other alternatives for non-experts. Interestingly, a model using history alone also had reasonable accuracy (86.1%). This result is better than expected, and may be due to the high proportion of focal neurological symptoms in PCS patients in our dataset, as well as the fact that our history data were collected by an expert clinician. Thus, this finding should be interpreted with caution, and will need to be reproduced using larger external datasets collected by non-experts.

Our results also suggest that the variables included in our tiers could be reduced and/or modified without impacting performance. In particular, it appeared that oVEMP could be omitted from our Tier 1 model; our presumptive clinical explanation for this is that whilst the majority of AUVP patients have an abnormal oVEMP, these patients would likely already be identifiable by their results from VNG and VHIT; in particular, almost all AUVP patients have an abnormal horizontal canal VHIT [16, 44]. Reducing the number of features required by our model has many advantages, and this will be one of the goals for our future work. Our results also indicate that VNG may be able to substitute for VHIT in our Tier 2 model. VNG is easier to perform than VHIT, although we note that our VNG data were collected by an expert examiner, and non-experts may have inferior results in the absence of nystagmus traces. Nevertheless, this holds promise for another model which would be accessible to non-experts.

### Clinical implications

In contemporary neuro-otological evaluation, HINTS constitutes a robust algorithm for evaluating AVS and is effective when performed by experts (as supported by our results). However, many clinicians assessing AVS are not experts at HINTS and often perform and/or interpret it incorrectly (particularly the head impulse test component [45]), resulting in unreliable findings. Our Tier 2 machine learning model with horizontal canal VHIT achieved the same accuracy as HINTS without requiring the same information, demonstrating its potential as an alternative diagnostic tool to address this practice gap for clinicians not confident with HINTs. The horizontal canal VHIT can be performed at the bedside in 5 min and is well tolerated even in acutely symptomatic patients [46, 47]; it also provides an objective, quantified result which is easier to interpret than the bedside equivalent, particularly if there is vigorous spontaneous nystagmus present (as is often the case in AUVP). Whilst VHIT does also require training to perform, this could be assigned to dedicated clinician(s) (who could be a technician or from allied health, and not necessarily the treating clinician). This means that history, bedside examination and VHIT (or perhaps VNG, as discussed above) could potentially be applied early in the diagnostic pathway (either in lieu of or in addition to HINTS [48, 49]) to provide rapid risk stratification prior to time-sensitive decisions regarding acute stroke therapy, potentially reducing unnecessary neuroimaging as well as complications from reperfusion therapies.

### Comparison with earlier studies

A previous study [21] also investigated the utility of machine learning models for differentiating PCS and AUVP in AVS and compared them against other classification methods including HINTS. Their best model used a novel deep learning algorithm and achieved 82% median accuracy, which was higher than HINTS (72.7%). The authors noted that HINTS performed worse in their cohort than expected from the literature, which may suggest that their dataset was harder to classify and makes comparison to our results difficult. Our study differs in our use of a larger patient dataset, as well as our focus on developing models tailored to specific clinical settings through our use of limited variable subsets; in contrast, their models used all 305 variables from history, health-related quality-of-life questionnaires (such as the Dizziness Handicap Inventory) and investigations including quantitative vestibular and oculomotor assessment, which together are beyond the capacity of an ER assessment. Similar to the feature importance scores from our all data model, they also found spontaneous nystagmus direction and SPV to be amongst the most influential variables, although their 2 most important variables came from VHIT (perhaps because their models did not use data from the bedside head impulse test).

### Limitations

A limitation of our study is that our history and examination was performed by an expert. Although our use of a structured digital history with standardised answers should improve reproducibility, history data may be less accurate if collected by a non-expert. The same applies to the examination findings, including the components of what we considered a basic examination; for example, an expert examiner who does not find typical features of AUVP on eye exam and bedside head impulse test may assess more carefully for focal weakness and ataxia to support a suspicion of PCS. In addition, our HINTS classifier also relied on nystagmus direction determined by VNG, which may have overestimated its accuracy. Our use of VNG to collect all nystagmus data, whilst best practice, denied us the opportunity to include naked eye nystagmus examination in our models, which would have improved generalisability. Generalisability is also limited by our use of custom software to obtain comprehensive metrics from VHIT beyond what the commercial software provides; as we refine our models to require fewer features, we hope to be able to demonstrate that the data from the commercial software will be sufficient. Thus, future work will evaluate model performance using data collected by a non-expert, without VNG and using VHIT metrics from the commercial software.

We recognise that there is an updated version of the HINTS algorithm called HINTS plus, which adds bedside screening for new hearing loss as a red flag for PCS in patients who would otherwise be classified as AUVP [50]. We did not perform this step for patients in this study; instead, all suspected AUVP patients received a bedside audiogram, as this was readily available at our institution. Our approach provides more objective assessment for hearing loss, but as a result we did not have bedside hearing assessment data to allow comparison of our models to HINTS plus. We also acknowledge that there are established clinical scores accessible to non-experts in AVS, in particular the graded gait/truncal instability (GTI) score, which can achieve 71% sensitivity and 83% specificity for central causes in AVS and outperforms the ABCD2 score [51]. It would have been worthwhile to compare our Tier 3 model to the GTI score on our patients, but unfortunately, we did not collect the gait/truncal ataxia data in the format for the GTI score. This will be of value in future evaluation of our models.

Another limitation of our study is that most patients with AUVP did not have a delayed MRI. Although MRI was done if uncharacteristic findings were present, such as skew or atypical results on vestibular testing, it is possible that some AUVP patients in our dataset had an incorrect gold-standard diagnosis, particularly as AUVP lacks a gold-standard ground truth test. We did not include vestibular migraine patients in this study, which can account for up to 10% of AVS presentations [52], but we plan to include them in subsequent studies. Future studies will also incorporate more sociodemographic data to evaluate for possible biases. Finally, we did not use an external dataset to evaluate our models, which is important to demonstrate generalisability and real-world applicability. The large number of variables we considered and the differences in workflow between institutions made it difficult to obtain matching datasets from other clinical sites, but this should be possible in the future as we refine our models to require fewer variables. External evaluation data will also allow for meaningful model calibration in future iterations.

We also acknowledge that this is a proof-of-concept study and does not include the final step of model deployment. Before model implementation, multi-centre trials and validation are needed to confirm generalisability across clinical settings, and the desired decision threshold for PCS must be determined Subsequently, additional considerations including optimising clinician adherence (e.g. user interface, offline functionality, and incorporation into ER workflow), addressing ethical risks (e.g. to ensure patient consent and data privacy), and establishing processes for ongoing monitoring and refinement must be addressed to ensure sustained benefits for clinicians and patients.

## Summary

Our study demonstrated that machine learning models can accurately separate AVS patients into PCS or AUVP based on information from history, examination and vestibular function tests. In particular, models using only history, basic bedside examination and (if available) VHIT could provide diagnostic support to non-expert ER clinicians, thereby addressing an important gap in current clinical practice.

## Supplementary Information

Below is the link to the electronic supplementary material.

Supplementary file1 (XLSX 22 KB)

Supplementary file2 (XLSX 14 KB)

Supplementary file3 (XLSX 18 KB)

Supplementary file4 (PDF 787 KB)

Supplementary file5 (XLSX 15 KB)

## Data Availability

The study dataset is not publicly available as it contains patient health data. A deidentified version may be made available to collaborating researchers via a data sharing agreement on reasonable request to the corresponding author M.W.

## Code availability

The code is similarly available on reasonable request.
