# Supplementary material for: Separating stroke and acute unilateral vestibulopathy using history, examination and vestibular tests: a machine learning approach
Source: J Neurol. 2026 Sep 8;273(10):573. doi: 10.1007/s00415-026-13779-0 (PMC13553692; doi:10.1007/s00415-026-13779-0)
Supplement: Supplementary file 1 [file 415_2026_13779_MOESM4_ESM.pdf]

Article Title: Separating Stroke and Acute Unilateral Vestibulopathy using History, Examination and Vestibular Tests: A Machine Learning Approach

Authors: Chao Wang, Kunal Chaturvedi, Benjamin Nham, Nicole Reid, Andrew P. Bradshaw, Sally M. Rosengren, Deborah A. Black, Kendall J. Bein, G. Michael Halmagyi, Mukesh Prasad, Ali Braytee, Gnana K. Bharathy, Miriam S. Welgampola

Journal: Journal of Neurology

Corresponding Author: Miriam S. Welgampola; Central Clinical School, University of Sydney, Australia; miriam@icn.usyd.edu.au

---

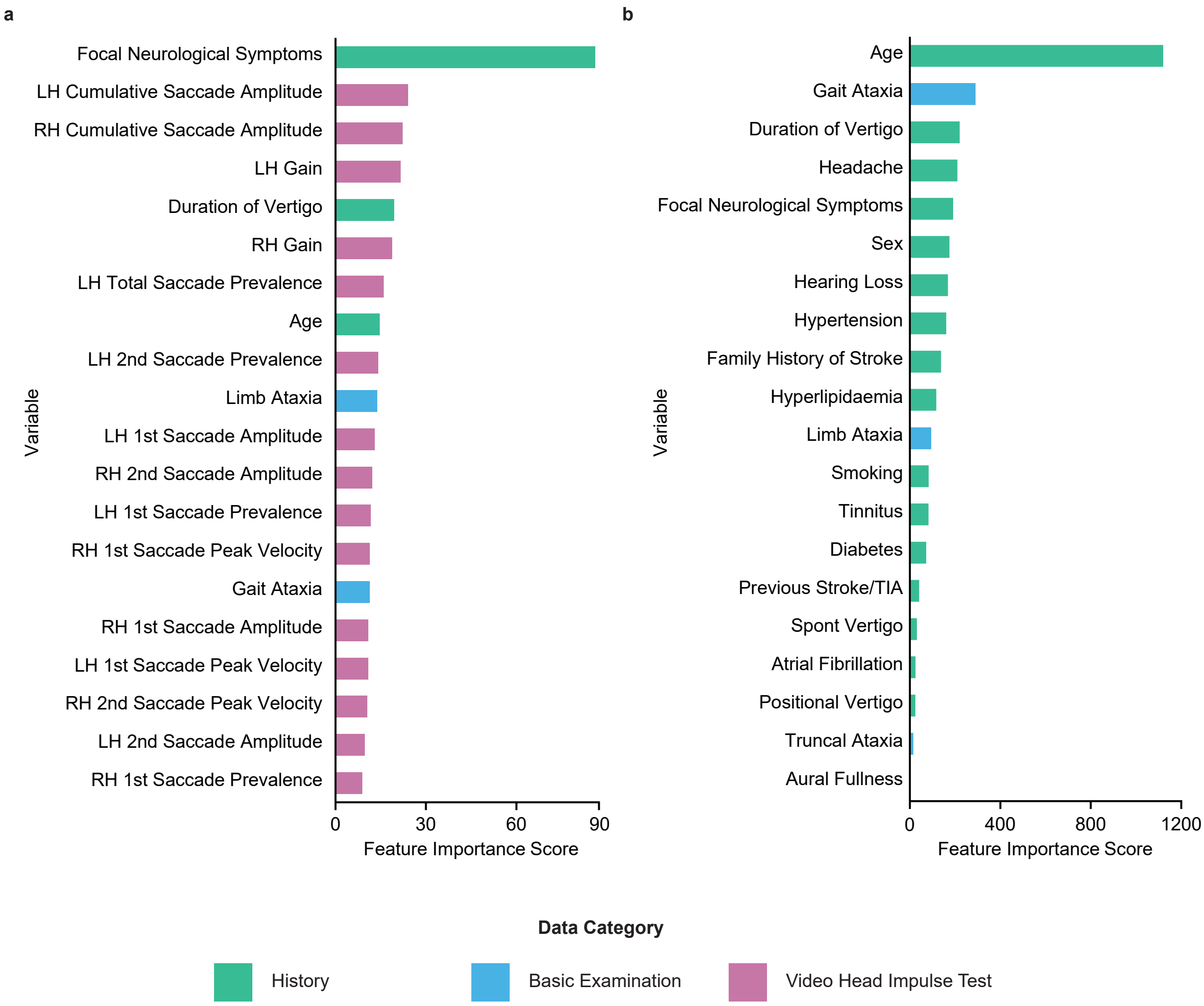

Online Resource 4. Top 20 variables with highest feature importance scores for the best models in Tiers 2 and 3. a Tier 2 (CatBoost): History, basic examination, horizontal canal video head impulse test. **b** Tier 3 (XGBoost): History and basic examination. A higher score indicates greater importance of the variable for model prediction. Colours indicate the data category which the variable is from. LH = left horizontal canal, RH = right horizontal canal, Spont = spontaneous, TIA = transient ischemic attack
